# Systematization of the Introduction of IPV and Switch from tOPV to bOPV in the Americas

**DOI:** 10.1093/infdis/jiw557

**Published:** 2017-06-30

**Authors:** Cristina Pedreira, Elizabeth Thrush, Barbara Jauregui

**Affiliations:** 1 Comprehensive Family Immunization Unit, Pan American Health Organization, and; 2 Independent Consultant for the Pan American Health Organization, Washington, DC

## Abstract

The synchronized introduction of the inactivated polio vaccine (IPV) and the switch from trivalent oral polio vaccine (tOPV) to bivalent oral polio vaccine (bOPV) has constituted an effort without precedents, and with astonishing results. Within the established time frame, all countries in our region managed to carry out the decision, planning, and introduction of this vaccine and subsequent switch to their national immunization schedules.

The purpose of this article is to systematize the process of IPV introduction and switch in Latin America and the Caribbean, which constitutes an important piece in the documentation of the polio legacy in the Americas. Regional level as well as country perspectives and viewpoints are described. Analyzing and summarizing the lessons learned from the introduction of IPV and the switch from tOPV to bOPV can be useful for the introduction of new vaccines in the Pan American Health Organization (PAHO) region and in other regions of the world, and to help our own region successfully carry out another synchronized vaccine introduction in the future, if necessary.

The purpose of this article is to share the experience of IPV introduction and switch in Latin America and the Caribbean. We conducted a descriptive analysis of PAHO support to countries for IPV introduction and the switch from tOPV to bOPV, and present the main findings on the countries experience around IPV introduction and the switch. This information constitutes an important piece in the documentation of the polio legacy in the Americas.

## BACKGROUND

Worldwide, sustained use of polio vaccines has led to a precipitous drop in the global incidence of poliomyelitis by over 99%, and the number of countries with endemic polio dropped from 125 to just 3 (Afghanistan, Pakistan, and Nigeria) in 2016, when only 28 cases were reported as of November [1, 2].

The Region of the Americas (AMR) was the first region of the World Health Organization (WHO) to establish the goal to eradicate polio and to achieve polio elimination. The last case of this disease caused by wild poliovirus in the AMR was detected in 1991 in Peru, and in 1994 the AMR was declared polio-free [3–5].

Following polio control in the AMR, in 1988 the 41st World Health Assembly (WHA) adopted the global goal to eliminate polio by the year 2000 [6]. By 2010–2011, all regions of the world except for the Americas had suffered poliovirus outbreaks in previously polio-free countries [7], and the world was not on track to interrupt poliovirus transmission [8]. For this reason, in May 2012, the 65th WHA declared the completion of poliovirus eradication a “programmatic emergency for global public health,” and a comprehensive strategic plan for the polio eradication endgame (the “Endgame Plan”) was approved in January 2013 [9].

Unlike previous plans, the Endgame Plan anticipates the eradication of all poliovirus: wild, vaccine-derived poliovirus, and Sabin virus. To do this, the use of oral polio vaccine (OPV) must be phased out. This phased withdrawal began in April 2016 with the global switch from the trivalent oral polio vaccine (tOPV), containing all 3 types of poliovirus, to the bivalent oral polio vaccine (bOPV), containing only types 1 and 3 [10, 11].

In order to ensure continued immunity against poliovirus type 2 after the switch, it was recommended that all countries introduce at least 1 dose of the inactivated poliovirus vaccine (IPV) before the switch [12, 13].

## THE ENDGAME PLAN IN THE AMERICAS

The Pan American Health Organization (PAHO) Technical Advisory Group of Immunization (TAG) supported the renewed polio eradication efforts and the endgame eradication goals, including the introduction of at least 1 dose of IPV and the eventual withdrawal of OPV from routine vaccination programs. However, different than the other WHO regions, the TAG recommended IPV as the first dose, in a sequential schedule of IPV followed by OPV, based on the epidemiology of the AMR [14, 15]. This schedule is most beneficial for the AMR given the fact that around 50% of vaccine-associated paralytic polio cases in the AMR are associated with the first OPV dose [16].

Prior to 2013, 32 countries in the AMR only had an OPV schedule. Between early 2015 and early 2016, all of these countries were able to introduce at least 1 dose of IPV into their routine schedules before the switch, and between 17 April and 1 May 2016, 36 countries in the AMR participated in the global switch from tOPV to bOPV.

## PAHO’S TECHNICAL SUPPORT TO COUNTRIES FOR IPV INTRODUCTION AND tOPV TO bOPV SWITCH IN THE AMR

**Figure 1.**
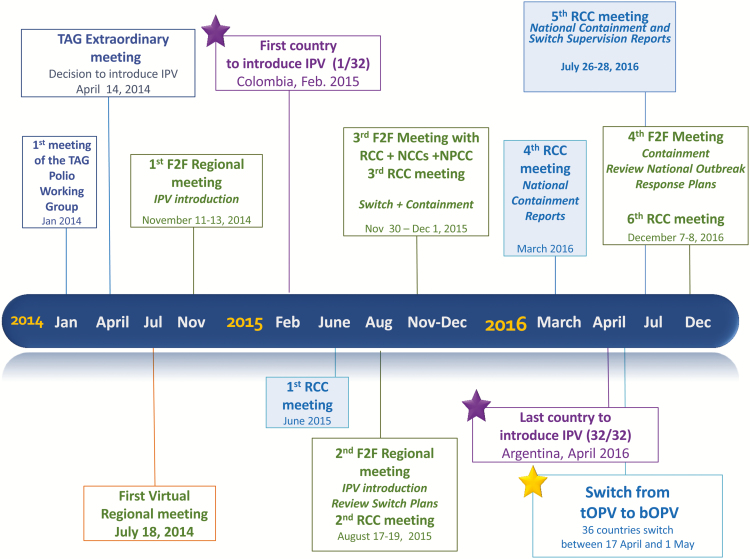
Timeline of events relevant to IPV introduction and switch in the Americas.

Based on TAG’s recommendation and the urgency of the IPV introduction and the switch, PAHO developed a comprehensive technical cooperation strategy, including several virtual and face-to-face meetings and the development and adaptation of support documents to maximize chances of a successful regional IPV introduction and switch. PAHO also maintained permanent and close contact with the countries, with absolute availability for communications and country missions as requested. Figure 1 shows a timeline with relevant regional events.

By the first quarter of 2015, PAHO had received the formal commitment from all Latin America and the Caribbean countries for the introduction of IPV.

To promote the implementation of uniform technical guidelines, training materials and communication messages across the AMR, PAHO developed the *PAHO IPV Introduction Practical Guide* [17] and adapted and expanded on several materials developed by the Immunization Management Group (IMG) of the Global Polio Eradication Initiative (GPEI), to support countries with IPV introduction [18]. (The IMG is made up of partners from WHO, United Nations Children’s Fund [UNICEF], Task Force for Global Health, GAVI [Global Alliance for Vaccines and Immunization], US Centers for Disease Control and Prevention [CDC], the Rotary Club, and the Bill and Melinda Gates Foundation [BMGF].) These materials were shared with countries in editable formats so they could be adapted as needed. Regarding the PAHO support to countries for the switch, there was no need to adapt the GPEI global guidelines for the switch; instead, countries adopted it with minor modifications [19].

By September 2015, PAHO had received and reviewed the switch plans from all countries to ensure that all key components had been included. PAHO created and maintained a dashboard to follow the whole process of the switch in the countries, which was monitored by the Regional Certification Commission (RCC) and National Certification Committees (NCCs). Additionally, PAHO provided significant direct technical cooperation, including visits to selected countries, to ensure preparedness and avoid any switch delays.

## OTHER PARTNERS’ ROLES AND CONTRIBUTIONS IN THE AMR

The remarkable success for the introduction of IPV and the switch in the AMR would not have been possible without the support from many international and regional partners. The support received from partners such as WHO Headquarters, UNICEF, CDC, the Task Force for Global Health, and GAVI was critical throughout the entire process of IPV introduction and the switch. These agencies provided valuable support to the AMR, including technical and/or financial support for the decision-making, planning, preparation, implementation, and validation of IPV introduction and the switch. The UNICEF Regional Office for Latin America and the Caribbean played a role in advocacy, social mobilization, and switch preparation and validation.

Financial support from multiple international sources was channeled through GAVI and GPEI for some countries. These funds supported gaps in the national budget for IPV introduction and the switch, principally for coordination, planning and preparation, social mobilization, advocacy, trainings, human resources, and evaluation. Additionally, some countries reported having received technical and financial support from other partners, such as the Rotary Club, that played an important role advocating for IPV introduction and participating in the independent monitoring of the switch.

Prior to the decision to introduce IPV in the AMR, the BMGF conducted an immunological study of 1 dose of IPV in Chile, and WHO supported studies of OPV-IPV in Cuba, which served as a key pieces of evidence to support the decision-making process in the AMR.

## DESCRIPTIVE ANALYSIS OF IPV INTRODUCTION PROCESS IN THE AMR

**Figure 2.**
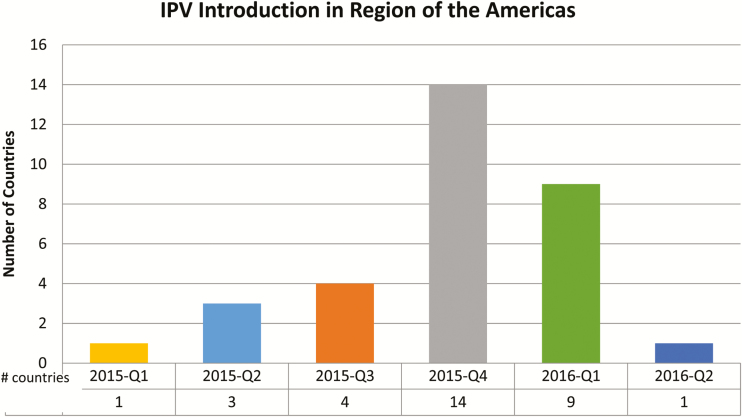
Number of countries that introduced IPV per quarter.

In the PAHO Region, 19 countries and territories, representing 70% of the birth cohort in the AMR, were already using the IPV vaccine in their national schedule prior to 2015. The remaining 32 countries, representing 30% of the birth cohort in the AMR (4606700) introduced IPV as part of the Endgame Plan, between 2015 (22 countries) and the first half of 2016 (10 countries; Table 1). See Figure 2 for the number of countries that introduced IPV per quarter.

**Table 1.** Polio Vaccination Schedules for 2014, 2015, and 2016 by Country

Country	Birth Cohort, 2015	Vaccination Schedule— Under 1 Year	
	(thousands)	2014	2015–2016
Anguilla	0.21	OPV	IPV + OPV
Antigua and Barbuda	1.47	OPV	IPV + OPV
Argentina	753.43	OPV	IPV + OPV
Aruba	1.41	Penta	Penta
Bahamas	5.83	OPV	IPV + OPV
Barbados	3.45	OPV	IPV + OPV
Belize	8.19	OPV	IPV + OPV
Bermuda	0.8	Penta	Penta
Bolivia	253.25	OPV	IPV + OPV
Bonaire	0.16	Hexa	Hexa
Brazil	3015.95	IPV + OPV	IPV^a^
Canada	386.74	Penta	Penta
Cayman Islands	0.68	Penta	Penta
Chile	234.23	OPV	IPV + OPV
Colombia	746.63	OPV	IPV + OPV
Costa Rica	69.83	Penta	Penta
Cuba	114.73	OPV	IPV + OPV
Curaçao	2.05	OPV	IPV + OPV
Dominica	1.13	OPV	IPV + OPV
Dominican Republic	215.84	OPV	IPV + OPV
Ecuador	330.81	OPV	IPV + OPV
El Salvador	105.3	OPV	IPV + OPV
French Guiana	6.67	Penta	Penta
Grenada	1.77	OPV	IPV + OPV
Guadeloupe	6.05	Penta	Penta
Guatemala	437.65	OPV	IPV + OPV
Guyana	14.82	OPV	IPV + OPV
Haiti	263.26	OPV	IPV + OPV
Honduras	168.91	OPV	IPV + OPV
Jamaica	48.15	OPV	IPV + OPV
Martinique	4.37	Penta	Penta
Mexico	2345.8	Penta	Penta^a^
Montserrat	0.06	OPV	IPV + OPV
Nicaragua	121.24	OPV	IPV + OPV
Panama	75.13	Hexa	Hexa^a^
Paraguay	140.62	OPV	IPV + OPV
Peru	614.68	IPV + OPV	IPV + OPV
Puerto Rico	43.23	IPV	IPV
Saba	0.017	Hexa	Hexa
Saint Kitts and Nevis	0.7	OPV	IPV + OPV
Saint Lucia	2.25	OPV	IPV + OPV
Saint Vincent and the Grenadines	1.39	OPV	IPV + OPV
Saint Maarten	0.53	Penta	Penta
St. Eustatius	0.036	Hexa	Hexa
Suriname	9.74	OPV	IPV + OPV
Trinidad and Tobago	18.98	OPV	IPV + OPV
Turks and Caicos Islands	0.81	OPV	IPV + OPV
United States	4024.58	IPV	IPV
Uruguay	48.57	IPV	IPV
Venezuela	599.43	OPV	IPV + OPV
Virgin Islands (UK)	0.37	OPV	IPV + OPV
**Total Birth Cohort**	**15251.72**		

Source: Country data sent to PAHO/WHO.

Abbreviations: Hexa, hexavalent; IPV, inactivated polio vaccine; OPV, oral polio vaccine; Penta, pentavalent.

^a^Countries that use OPV as booster dose.

Eleven countries had initially planned to introduce more than 1 dose of IPV, but due to global vaccine shortage, the PAHO Immunizations team finally recommended that all countries introduce only 1 dose of IPV, until an adequate global supply of vaccine became available.

In March 2016, PAHO sent out a survey to the 32 countries from Latin American and the Caribbean that introduced IPV in 2015 or 2016 as part of the Endgame Plan. The survey was sent to each country via the PAHO country office with the request that the Expanded Program on Immunization (EPI) team complete and return the survey to PAHO within 1 month.

Thirty-one out of the 32 countries replied to the survey. Each country’s answers were read individually in their original language to become familiar with the perceptions of each country before coding. Key ideas were highlighted for each response. Once all responses were received, read, and translated to English, a matrix was created, which listed the questions in rows and each country’s response in columns.

Some countries mentioned the same topic multiple times in different answers of their survey. In an effort to avoid swaying the results for the important facilitators and barriers in the AMR, a table was created where countries were counted only once, independent of how many times they mentioned the same topic in their survey response.

A summary of the results is presented in this section (refer to Table 2 for the main findings). An important limitation worth noting is the potential for recall bias and other sources of bias between the countries that have introduced the vaccine recently and the ones that introduced the vaccine a year ago or more. Another limitation is that the survey reflects the viewpoints of the EPI manager only, not all of the EPI and other staff involved in the IPV introduction effort.

**Table 2:** Main Findings of the IPV Introduction Survey from Countries

Dimensions		IPV Introduction Survey Key Findings for Each Country (N=31)	No. of Countries	Percent
Decision to introduce IPV	Time to decide	Countries that took 6 months or less to make decision	26	86
		Countries that took 1–3 months to make decision	17	56
	Main facilitators	Global commitment	9	29
		National political support and commitment	6	19
		Presence of a regional TAG recommendation	5	16
		Availability of supporting evidence regarding rationale for the introduction	4	13
	Main barriers	No difficulties in the decision-making process	21	68
		Financial issues	4	13
IPV introduction process itself	Nationwide or phased introduction	Countries that introduced IPV simultaneously nationwide	25	81
		Countries with phased introduction	6	19
	Main facilitators	PAHO support (technical cooperation and guidelines)	23	74
		Staff training	19	61
		Political will and support	17	55
		Commitment of staff	17	55
		International commitment to the need for global IPV introduction to achieve polio eradication	14	45
		Experience, preparedness, and planning of the EPI	13	42
	Main barriers	Negative perception of change from drop to shot administration	19	61
		Insufficient or delayed training	12	39
		Financial constraints	8	26
		Insufficient monitoring or supervision in the field	8	26

Abbreviations: EPI, Expanded Program on Immunization; IPV, inactivated polio vaccine; PAHO, Pan American Health Organization; TAG, Technical Advisory Group of Immunization.

### Decision-making Process for IPV Introduction

**Figure 3.**
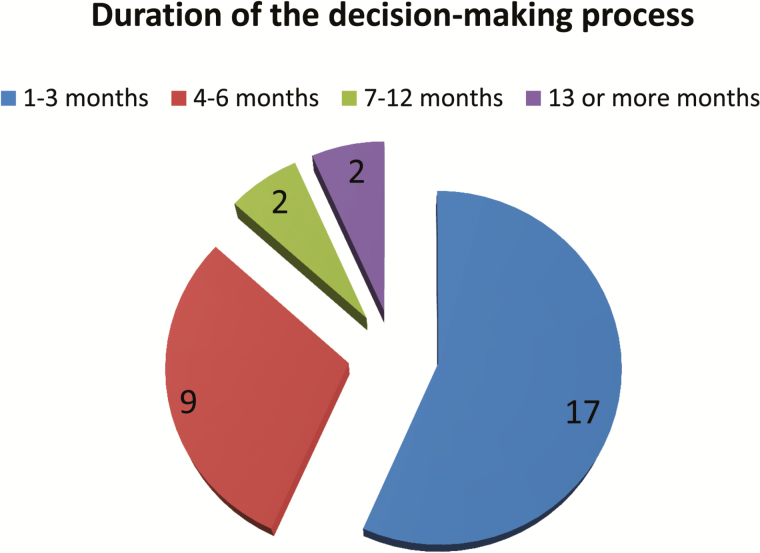
Duration of decision-making process.

Countries adopted the vaccine with unprecedented speed after the Regional TAG recommendation: 26/31 of the countries (86%) took 6 months or less to make the decision to introduce IPV, and an impressive amount of 17/31 countries (56%) took 1 to 3 months (Figure 3).

The immunization program alone initiated the decision-making process in half of the countries (15/31), and in the other half (14/31), both the immunization program and the Ministry of Health coinitiated the process. Not surprisingly, almost all respondent countries (29/31) referred that the Ministry of Health authorities had the final say in the decision of introducing IPV. Only 3/31 countries said that the Presidency and/or Ministry of Finance were involved in the final say.

It is noteworthy that, even though there was a WHA mandate to introduce the vaccine and a Regional TAG recommendation endorsing the introduction, countries still felt the need to consult their National Immunization Technical Advisory Group (NITAG). Fourteen of the 17 non-island countries involved their NITAG in the process, whereas the Caribbean discussed the issue at their EPI managers meeting, which resulted in a subregional endorsement of the introduction of IPV and the switch.

Surprisingly, the majority of the countries (21/31) did not record any difficulties in the decision-making process. Only 4 countries mentioned financial issues as a complication in the decision-making process.

Global commitment was the most frequently mentioned facilitating factor for the decision to introduce IPV (29%), followed by national political support and commitment (19%), the presence of a regional TAG recommendation (16%), and the availability of supporting evidence around the rationale for the introduction (13%).

The decision to introduce IPV had coverage in almost half the countries (14/31), with radio being the most popular medium followed by newspaper and television. Many countries that announced the decision in the media received a positive media opinion (9/14 countries), whereas in a few countries, either the media expressed concern about adding 1 more shot to the well-child visit (3/14 countries) or had a neutral opinion about the introduction (2/14 countries).

### Planning and Preparing for IPV Introduction

Two-thirds of the countries (20/31) did not need to make changes to their EPI infrastructure in preparation for the introduction of IPV, while one-third (11/31) needed to make changes. The changes that were required included expanding the cold chain (6/11 countries) and updating the immunization records and report forms to include IPV (4/11 countries). One country did not specify the changes required.

**Figure 4.**
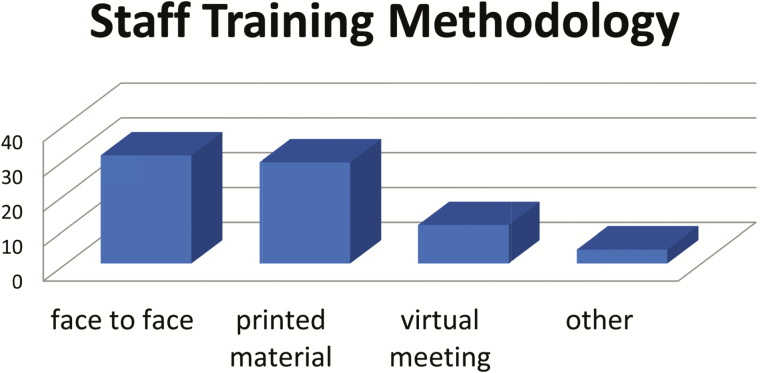
IPV introduction training methodology.

In order to prepare health-care workers for the introduction, all countries (31/31) used face-to-face trainings, and most (29/31) used printed materials as well. Additionally, one-third of the countries also conducted virtual meetings, and a few used other strategies, including teleconferences (Figure 4).

### Vaccine Introduction

Twenty-five of 31 countries introduced IPV simultaneously nationwide, while 6 countries introduced it in phases. Countries with phased introduction were: Barbados, Bolivia, Guyana, Haiti, St. Kitts, and Suriname.

For two-thirds of the countries (22/31), the introduction meant that children started to receive more than 2 injectable vaccines in a single visit.

Several countries (7/31) mentioned the Ministry of Health and the professional associations (6/31) as national entities that provided support to the decision-making process and introduction of IPV. Additionally, 24/31 countries mentioned PAHO and 12/31 mentioned other international and regional entities that provided support, including financial support.

### Communication of the Introduction

The general public received information through the radio (16/31 countries) and printed communication (13/31 countries), as well as television communications (7/31 countries) about the introduction of IPV. An overwhelming 77% (24/31 countries) replied that they did not perceive any challenges to communicate the introduction of IPV, and of the 7 who did mention challenges, 4 countries said that it was related to the change in IPV administration from a drop to a shot.

The public had a positive reaction to the introduction in two-thirds of the countries (22/31), with 9 of these countries expressing that the public had initial concern but after the communication efforts had embraced the change.

## LESSONS LEARNED FROM IPV INTRODUCTION IN THE AMR

### Facilitators of the IPV Introduction Process

Overall, the dominant themes within the facilitators were: commitment, engagement or buy-in from the different stakeholders, and knowledge. The support that PAHO provided to countries, either through technical cooperation or dissemination of guidelines and other materials, was the single most prominent facilitator for the regional introduction of IPV, mentioned by over two-thirds of the countries (23/31 countries). Other important positive factors were staff training (19/31); political will and support (17/31); commitment of staff (17/31); international commitment to the need for global IPV introduction to achieve polio eradication (14/31); and experience, preparedness, and planning of the EPI (13/31). Several other facilitators were mentioned.

### Difficulties in the IPV Introduction Process

The majority of difficulties that were mentioned were unique to just 1 or 2 countries. The most common identified barrier for IPV introduction was the negative perception of the change in administration from drops to a shot (19/31). For most countries (23/31), the addition of IPV to the routine immunization schedule meant 3 or more injections at a single visit for a 2-month-old. Nonetheless, many of the countries that expressed this concern (9/31) also stated that after careful communication messaging and training of health-care workers, both the public and the staff felt reassured, and in the end, the public had a positive reaction to the introduction in two-thirds of the countries (22/31).

Insufficient or delayed training was mentioned as the second most common difficulty (12/31 countries), which reinforces the notion that staff training played a pivotal role in the success of IPV introduction.

Financial constraints were also expressed by 8/31 countries as a factor that hindered the IPV introduction process. Five countries were able to receive external financial support for IPV introduction, while the remaining 3 did not receive external financial support, but they still introduced the vaccine. This talks to the commitment of the countries of the AMR toward immunization in general, and more specifically toward polio elimination.

Insufficient monitoring or supervision in the field was also mentioned by several countries (8/31), which prevented them from evaluating the successful implementation of the program.

### Lessons Learned for the Future: What Countries Would Do Different in the Future

Ten out of the 31 countries did not mention anything that they would do different. The rest (21/31) replied with a variety of things they would change, including:

- Increase communication about the introduction to doctors in the private sector and to other stakeholders (6/31)
- Enhance supervision activities (5/31)
- Strengthen the training (4/31)
- Conduct earlier, better planning (3/31)

### Countries’ Comments About the Materials Provided

Ninety percent of countries (28/31) used the IPV Introduction Guide provided to them, and almost all (27/31) said this was very useful. Ninety percent of the countries also used the background and technical rational documents, and 22/31 reported they were very useful as well. Around 70% of the countries used the training modules that were developed, and reported that they were also very useful.  

### National, Regional, and International Support That Would Have Been Useful But Was Not Provided

Two-thirds of the countries (20/31) did not mention any type of support that would have been useful but was not provided, and the remaining 11 countries mentioned the need for more internal support, including PAHO country office being more present in the field (4/31), and more support for communication and dissemination (2/31).

## DESCRIPTIVE ANALYSIS OF THE SWITCH FROM tOPV TO bOPV IN THE AMR

Thirty-six countries of the AMR switched from tOPV to bOPV in April 2016. In July 2016, PAHO administered a survey to these 36 countries through the same channels as the previous survey; all countries replied, and PAHO analyzed the information received using the same methodology previously described. Table 3 provides a summary of the main findings.

**Table 3:** Main Findings from the Switch Survey from Countries

Survey Information		Switch Survey Key Findings for Each Country (N=36)	No. of Countries	Percent
Planning the switch	Main facilitators	Staff training	11	31
		Counting on PAHO technical support and documents	11	31
		Commitment of healthcare workers	9	25
		Involvement of healthcare workers and key national players	9	25
		Political will	7	19
	Main barriers	Countries that did not encounter any obstacles in the planning process	15	42
		Concomitant events as a factor that made the planning more difficult	11	31
Implementing the switch	Main facilitators	Commitment of healthcare workers	10	28
		Monitoring and supervision activities	5	14
		Staff training	4	11
	Main barriers	Countries with no implementation obstacles for the switch	14	39
		Vaccine transportation–related issues	7	19
Validating the switch	Main facilitators	Commitment/support of stakeholders involved in the validation process	12	33
		External support (technical or financial)	10	28
	Main barriers	Countries with no obstacles in the validation process	11	31
		Insufficient financial resources for the switch	5	14
		Delays in receiving the validation forms from the lower level	5	14

Abbreviation: PAHO, Pan American Health Organization.

### Process of the Switch From tOPV to bOPV

Almost all countries (35/36) had national-level switch coordination committees, while 13/36 also had regional-level committees, 10/36 had departmental-level committees, and 8/36 had municipal-level committees. Some countries used subcommittees, including logistics, containment, surveillance, and communications, among others. Slightly less than half of the countries (14/36) used an already existing committee for this purpose.

The most common departments of the Ministry of Health that were involved in the committee included Epidemiology (7), Public Health (6), and Surveillance (3), among others.

Most countries (25) indicated that other ministries did not participate in the committee. Some countries indicated participation in the committee from the Ministries of Education, Agriculture, Finance, Natural Resources and Environment, Defense, Interior, and Labor.

Twenty-three of 36 countries said there were actors from outside the government that participated in the committee, including private institutions and professionals, the Rotary club, professional associations, and universities and scientific societies, to name a few. Beyond participation in the committees, outside actors played a significant role in all stages of the switch process, including 19 countries that involved regional and international organizations, 10 that involved scientific societies, 6 that involved nongovernmental organizations (NGOs), and 3 that involved the Rotary Club.

**Figure 5.**
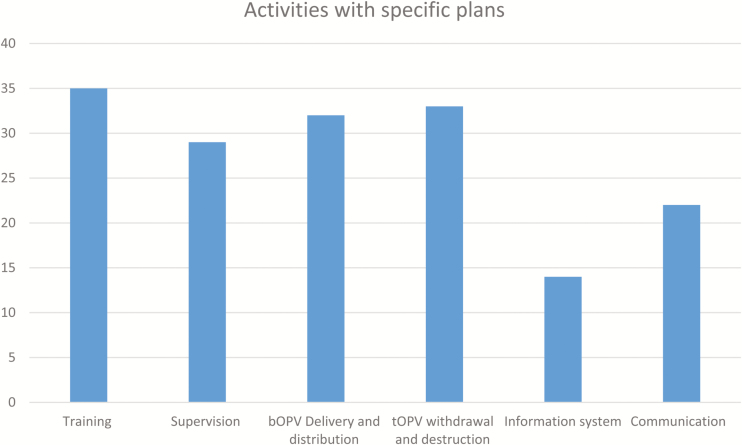
Activities with specific plans.

There were specific plans developed for a variety of activities, including training (35/36), bOPV delivery and distribution and tOPV withdrawal and destruction (33/36), supervision (29/36), communication (22/36) and information system (14/36) (Figure 5).

All countries used face-to-face meetings as the main training methodology, but they also frequently used a mixed methodology for training, including virtual trainings and printed materials. Over two-thirds of the countries (27/36) used cascade training to train at the different levels. The training materials used were Microsoft PowerPoint presentations (36/36) and printed materials (29/36); one-third of the countries used videos (14/36).

Almost half of the countries (17/36) made changes to their information system, to adapt it to the new schedule with the alerts and exceptions needed.

Over half of the countries (20/36) implemented supplementary vaccination activities in preparation for the switch. Of these, most did these supplementary activities across the whole country (16/20).

### bOPV-tOPV Exchange Logistics

Twenty-four of the 36 countries had bOPV delivered to health facilities before switch day. Nine countries delivered bOPV on the switch day. Three countries only use OPV in their campaign, so prior to the switch they withdrew all tOPV and will introduce bOPV in the next national campaign.

### Communicating the Switch

Twenty-nine of the 36 countries targeted different audiences with specific communication activities. Almost all countries targeted the health-care workers, and some countries also targeted parents and caregivers, the media, and the general public.

Almost all countries (34/36) conducted briefings with key stakeholders, such as pediatricians, medical associations, NGOs, and so forth, in advance of the switch. Half of the countries (17/36) stated having organized media or public communication activities, such as press releases. Also, half of the countries (17/36) had a risk communication or crisis communication plan in place. The most common communication materials used by countries were posters or brochures (18/36), press releases (11/36), and radio spots (10/36).

### Validation and Supervision of the Switch

All 36 countries validated the switch through independent monitoring. Thirty of the 36 countries implemented the validation of 100% of warehouses and 10% of vaccination services within the recommended 15-day period following the national switch date, and submitted validation reports to PAHO; the other 6 countries were able to complete the validation of the switch after 15 days. After the validation period, all 36 countries completed supervision of the switch by visiting 100% of warehouses and vaccination services within 3 months of the switch date with their regular staff. All country reports were reviewed by the NCC, and subsequently by the RCC (Table 4).

**Table 4:** Final Supervision of the Switch in the Region of the Americas

No. of Countries	Total Warehouses in Country (n)	No. of Warehouses where tOPV was Found		Total No. of Vaccination Services (n)	No. of Vaccination Services where tOPV was Found		bOPV or IPV Availability		tOPV Disposed	
		In Cold Chain	Outside w/o Label		In Cold Chain	Outside w/o Label	bOPV (%)	IPV (%)	No.	Method
36	6132	50	11	98253	220	31	95	93	5995247	…^a^

Abbreviations: bOPV, bivalent oral polio vaccine; IPV, inactivated polio vaccine; tOPV, trivalent oral polio vaccine.

^a^Most of the countries used incineration as a destruction method.

During these visits to the 6132 warehouses, 50 (0.8%) warehouses were found with tOPV in the cold chain, and 11 (0. 2%) with tOPV not properly labelled outside the cold chain. The visits to the 98253 vaccination services found that 220 still had tOPV in the cold chain and 31 had tOPV not properly labelled out of the cold chain. In total, 5995247 doses of leftover tOPV were destroyed. The most common method of destruction was incineration. Ninety-five percent of all service points that use bOPV in the routine program had the vaccine available. This figure excludes Brazil, Cuba, and Mexico, because these countries only use bOPV in their campaigns. Ninety-seven percent of all service points in the AMR had IPV available. Of the 3% of service points that did not have IPV, 2.5% of this figure relates to 1 country that, at the time of the survey, had issues with IPV supply.

### Obstacles Encountered in the Switch

Forty-two percent of the countries (15/36) replied that they did not encounter any obstacles in the planning process. Half of the countries that did encounter obstacles mentioned concomitant events as a factor that made the planning more difficult (11/21).

Thirty-nine percent of the countries (14/36) did not mention any implementation obstacles of the switch. Vaccine transportation–related issues (7/36) were the most frequent obstacle mentioned.

Finally, 30% of the countries (11/36) did not mention any obstacles in the validation process, whereas some countries mentioned insufficient financial resources to perform it (5/36) or reported delays in receiving the validation forms from a lower level (5/36).

Table 5 summarizes positive and negative aspects of the switch from the countries’ perspectives.

**Table 5:** Best Practices and Opportunities for Improvement during the Switch

**Best Practices during the Switch:**
The most frequently mentioned best practice during the switch in general was the commitment of health-care workers (19/36)
**Facilitators during the Planning Process:**
• Staff training (11/36)
• Counting on PAHO technical support and documents (11/36)
• Commitment of healthcare workers (9/36)
• Involvement of healthcare workers and key national players (9/36)
• Political will (7/36)
**Facilitators for the Implementation of the Switch:**
• Commitment of healthcare workers (10/36)
• Monitoring and supervision activities (5/36)
• Staff training (4/36)
**Facilitators for Switch Validation:**
• Commitment/support of stakeholders involved in the validation process (12/36)
• External support (technical or financial) (10/36)
**Things to Do Differently in the Future:**
When asked what they would have done differently, countries replied that they:
• Would have started planning earlier (5/36)
• Would have done more supervision (5/36)

Abbreviation: PAHO, Pan American Health Organization.

### Countries’ Perception of PAHO Support for the Switch

When asked how satisfied they were with PAHO’s support for the switch in general, all but 1 country replied. The 35 countries that replied had a positive view of PAHO’s support. Two-thirds of the respondent countries (21/35) considered PAHO’s support very good, and the remaining 14 countries considered it good. Countries that stated that the support from PAHO was “good” (and not “very good”) mentioned vaccine supply issues (3/14) and financial requests issues (2/14) as some of the problems faced with PAHO’s support.

The types of support that PAHO provided for the switch that were mentioned most by countries were:

- Direct support to countries (25/36)
- Documents and materials (16/36)

Countries were asked to rank different types of PAHO support by degree of importance. The guidelines and supporting documents received were deemed the most important type of PAHO support for the switch (20/36), and regional face-to-face meetings were the next most important PAHO support provided (12/36).

### Regional Perspective

Despite some manifestations of concern with the timeline and the budget required, the countries of the AMR always maintained a positive and readiness attitude throughout this process. This is, in part, thanks to the countries’ commitment to immunization in general, but also due to a tremendous commitment to polio eradication in particular, which has a mystique in itself. Countries of the AMR feel ownership and pride about polio eradication and want to see the end of this story.

One of the most critical points for the success of this unprecedented worldwide and regional effort was ensuring that the countries understood the rationale and scientific basis for the decision to introduce IPV and make the switch from tOPV to bOPV, and the risk if this did not occur.

Other facilitators were the experience of the AMR on new vaccine introduction, the experience with multiple injections in a single visit (which allowed health-care providers to be prepared for the challenge ahead), and the experience of several countries in the AMR with introduction and use of IPV.

Additionally, the PAHO Revolving Fund for Vaccine Procurement (RF), a cooperation mechanism for the joint procurement of vaccines, syringes, and related supplies, was a game changer for the AMR, because most countries (98%) readily accepted the vaccines without having to go through a special country registration as long as they are procured through the RF, so this had a positive impact in the introduction of IPV and bOPV.

With regard to challenges faced in the AMR, the problems with the global vaccine supply and vaccine delays were major obstacles that had to be dealt with both at regional and national levels. A sense of Pan Americanism (understood as the desire and need to work together toward a common goal) played an important role when the global vaccine shortage did not allow for countries to introduce more than 1 dose of IPV, and PAHO had to recommend all countries who were not already using IPV to introduce a single dose. Other challenges included the fact that some countries with a more centralized government had more difficulty in making the decision.

Finally, the global structure to support the regions played a crucial role in the AMR. As mentioned previously, the coordinated work of the partners, specifically through the work of the IMG, provided enormous support to countries throughout the whole process for both IPV introduction and the switch from tOPV to bOPV.

In the history of vaccine introductions, there has never been a vaccine that had to be introduced by so many countries in such a short time frame. Additionally, the simultaneous switch from tOPV to bOPV was a unique and unprecedented event in global public health history. Overall, what seemed impossible ended up being possible thanks to country ownership of the polio eradication goals, and the excellent coordination and contribution of all partners.

## Notes

***Acknowledgments.*** PAHO would like to thank all of the countries and the health-care workers for the amazing commitment and dedication to meet these goals. We also thank the EPI managers for sharing their experience and perspective to enrich this legacy document. Thanks to Anguilla, Antigua and Barbuda, Argentina, Bahamas, Barbados, Belize, Bolivia, Brazil, British Virgin Islands, Chile, Colombia, Cuba, Curacao, Dominica, Ecuador, El Salvador, Grenada, Guatemala, Guyana, Haiti, Honduras, Jamaica, Mexico, Montserrat, Nicaragua, Panama, Paraguay, Peru, Dominican Republic, St Kitts and Nevis, St Lucia, St Vincent and Grenadines, Suriname, Trinidad and Tobago, Turks and Caicos, and Venezuela.

***Supplement sponsorship.*** This work is part of a supplement coordinated by the Task Force for Global Health with funding provided by The Bill and Melinda Gates Foundation and the Centers for Disease Control and Prevention.

***Potential conflicts of interest.*** All authors: No reported conflicts.

All authors have submitted the ICMJE Form for Disclosure of Potential Conflicts of Interest. Conflicts that the editors consider relevant to the content of the manuscript have been disclosed. The authors are staff members or consultants of the Pan American Health Organization. The authors alone are responsible for the views expressed in this publication, and they do not necessarily represent the decisions or policies of the Pan American Health Organization.

## References

[1] Global Polio Eradication Initiative. Polio eradication and endgame strategic plan 2013–2018 executive summary. Geneva, Switzerland: World Health Organization, 2013:1–16.

[2] Global Polio Eradication Initiative. Polio this week, as of 13 September 2016 http://www.polioeradication.org/dataandmonitoring/poliothisweek.aspx Accessed 13 September 2016.

[3] Pan American Health Organization Directing Council CD33/12. Plan of action for the eradication of the indigenous transmission of the wild poliovirus. In: Provisional Agenda Item 5.2. Washington, DC: Pan American Health Organization, 1988:1–29.

[4] Pan American Health Organization. Immunize and protect your children. Expanded Program of Immunization Newsletter. Washington, DC: Pan American Health Organization, 1994; 16:1–8.

[5] de Quadros CA, Robbins FC Certifications of the eradication of indigenous transmission of wild poliovirus in the Americas. J. Infect Dis 1997; 175: Suppl 1:S281–5. 9203731 10.1093/infdis/175.supplement_1.s281

[6] World Health Assembly. Global eradication of poliomyelitis by the year 2000. In: WHA41.28. Geneva, Switzerland; World Health Organization, 1988 http://www.polioeradication.org/content/publications/19880513_resolution.pdf Accessed 23 September 2016.

[7] Global Polio Eradication Initiative. Wild Poliovirus list. http://polioeradication.org/polio-today/polio-now/wild-poliovirus-list/ Accessed 13 January 2017.

[8] World Health Organization. Meeting of the Strategic Advisory Group of Experts on immunization, November 2011 conclusions and recommendations. Wkly Epidemiol Rec 2012; 87:1–16. 22242233

[9] World Health Assembly. Poliomyelitis: intensification of the global eradication initiative. In: WHA65.5. Geneva, Switzerland; World Health Organization, 2013 http://apps.who.int/gb/ebwha/pdf_files/WHA65/A65_R5-en.pdf Accessed 23 September 2016.

[10] World Health Assembly. Poliomyelitis. In: WHA68.3. Geneva, Switzerland; World Health Organization, 2015 http://apps.who.int/gb/ebwha/pdf_files/WHA68/A68_R3-en.pdf Accessed 23 September 2016.

[11] World Health Organization. Cessation of use of trivalent oral polio vaccine and introduction of inactivated poliovirus vaccine worldwide, 2016. Wkly Epidemiol Rec 2016; 91:421–32. 27623614

[12] Garon J, Seib K, Orenstein WA Polio endgame: the global switch from tOPV to bOPV. Expert Rev Vaccines 2016; 15:693–708. 26751187 10.1586/14760584.2016.1140041

[13] World Health Organization. Introduction of inactivated polio vaccine and switch from trivalent to bivalent oral poliovirus vaccine worldwide, 2013–2016. Wkly Epidemiol Rec 2015; 90:337–48. 26151981

[14] Pan American Health Organization. Technical Advisory Group on Vaccines and Immunization Polio Working Group. Final Report. Washington, DC, 2014:19.

[15] Pan American Health Organization. Technical Advisory Group on Vaccines and Immunization (TAG) Extraordinary Meeting Report, April 2014.

[16] Landaverde JM, Trumbo SP, Danovaro-Holliday MC, Cochi SE, Gandhi R, Ruiz-Matus C Vaccine-associated paralytic poliomyelitis in the post elimination era in Latin America and the Caribbean, 1992–2011. J Infect Dis 2014; 209:1393–402. 24520126 10.1093/infdis/jit602

[17] Pan American Health Organization. Practical Guide: Inactivated poliovirus vaccine (IPV) introduction. Washington, DC: Pan American Health Organization, 2014.

[18] Pan American Health Organization. IPV introduction—supporting technical documents. Washington DC: Pan American Health Organization, 2016 http://www.paho.org/hq/index.php?option=com_content&view=article&id=10926&Itemid=1707&lang=en Accessed 19 September 2016.

[19] Pan American Health Organization. Switch from tOPV to bOPV—supporting technical documents. Washington DC: Pan American Health Organization, 2016 http://www.paho.org/hq/index.php?option=com_content&view=article&id=11015&Itemid=1707&lang=en Accessed 19 September 2016.

